# Dynamic simulation of red blood cell metabolism and its application to the analysis of a pathological condition

**DOI:** 10.1186/1742-4682-2-18

**Published:** 2005-05-09

**Authors:** Yoichi Nakayama, Ayako Kinoshita, Masaru Tomita

**Affiliations:** 1 Institute for Advanced Biosciences, Keio University, Tsuruoka, 997-0017, Japan

**Keywords:** kinetics, metabolism

## Abstract

**Background:**

Cell simulation, which aims to predict the complex and dynamic behavior of living cells, is becoming a valuable tool. In silico models of human red blood cell (RBC) metabolism have been developed by several laboratories. An RBC model using the E-Cell simulation system has been developed. This prototype model consists of three major metabolic pathways, namely, the glycolytic pathway, the pentose phosphate pathway and the nucleotide metabolic pathway. Like the previous model by Joshi and Palsson, it also models physical effects such as osmotic balance. This model was used here to reconstruct the pathology arising from hereditary glucose-6-phosphate dehydrogenase (G6PD) deficiency, which is the most common deficiency in human RBC.

**Results:**

Since the prototype model could not reproduce the state of G6PD deficiency, the model was modified to include a pathway for *de novo *glutathione synthesis and a glutathione disulfide (GSSG) export system. The *de novo *glutathione (GSH) synthesis pathway was found to compensate partially for the lowered GSH concentrations resulting from G6PD deficiency, with the result that GSSG could be maintained at a very low concentration due to the active export system.

**Conclusion:**

The results of the simulation were consistent with the estimated situation of real G6PD-deficient cells. These results suggest that the *de novo *glutathione synthesis pathway and the GSSG export system play an important role in alleviating the consequences of G6PD deficiency.

## Introduction

Many attempts have been made to simulate molecular processes in cellular systems. Perhaps the most active area of cellular simulation is the kinetics of metabolic pathways. Various software packages that quantitatively simulate cellular processes and are based on numerical integration of rate equations have been developed. These include GEPASI [1], which calculates steady states as well as reaction time behavior; V-Cell [2], a solver of non-linear PDE/ODE/Algebraic systems that can represent the cellular geometry; and DBsolve [3], which combines continuation and bifurcation analysis.

The E-Cell project [4,5], which aims to model and simulate various cellular systems, was launched in 1996 at Keio University. The first version of the E-Cell simulation system, a generic software package for cell modeling, was completed in 2001. E-Cell version2, which is a Windows version of the first E-Cell system, is now also available [6]. E-Cell version 3, which enables multi-algorithm simulation, is the latest version [7]. The E-Cell system allows the user to define spatially discrete compartments such as membranes, chromosomes and the cytoplasm. The collections of molecules in all cellular compartments are represented as numbers of molecules, which can be converted to concentrations, and these can be monitored and/or manipulated by employing the various graphical user interfaces. In addition, the E-Cell system enables the user to model not only deterministic metabolic pathways but also other higher-order cellular processes, including stochastic processes such as gene expression, within the same framework. By using the E-Cell system, a virtual cell with 127 genes that are sufficient for "self-support" [4] was developed. This gene set was selected from information about *Mycoplasma genitalium *genomic sequences and includes genes for transcription, translation, the glycolysis pathway for energy production, membrane transport, and the phospholipid biosynthesis pathway for membrane production.

On the basis of existing models of single pathways and enzymes, various in silico models of human red blood cell (RBC) metabolism were first developed by Joshi and Palsson [8-11]. Subsequently, other groups developed RBC models [12-15]. The RBC is thought to be a good target for biosimulation because extensive studies over the last three decades have generated extensive biochemical data on its enzymes and metabolites. Moreover, the RBCs of many species, including humans, do not contain a nucleus or carry genes. This means that gene expression can be excluded from the model, which greatly simplifies the biosimulation. RBCs take up glucose from the plasma and process it by glycolysis, which generates the ATP molecules that are used in other cellular metabolic processes. The ATP molecules are mostly consumed by the ion transport systems that maintain the osmotic balance of the cell.

Here we describe our computer model of the human RBC, which we developed on the basis of previous models [8-13]. Our prototype model of the human RBC consisted only of glycolysis, the pentose phosphate pathway, nucleotide metabolism and simple membrane transport systems such as the Na^+^/K^+ ^antiport channel. Here, we have employed this prototype model to reproduce the pathological condition of glucose-6-phosphate dehydrogenase (G6PD) deficiency. This is the most common hereditary enzyme deficiency in RBCs; it causes anemia, and more than 400 varieties of G6PD deficiency have been identified [16]. The deficiency is known to exert only mild effects as it does not cause clinically significant problems in most cases, except upon exposure to medications and foods that cause hemolysis. Computer simulations for analyzing this deficiency have been reported [17-19], but these simulation models consisted only of glycolysis and the pentose phosphate pathway. We found that including the glutathione (GSH) biosynthesis pathway and the glutathione disulfide (GSSG) export system, which are involved in suppressing oxidative stress, improved the ability of the model to reflect the real diseased RBC. This suggests that these pathways may compensate for the consequences of G6PD deficiency in human RBCs.

## Methods

### Development of the prototype model and simulation experiments

**Figure 1:**
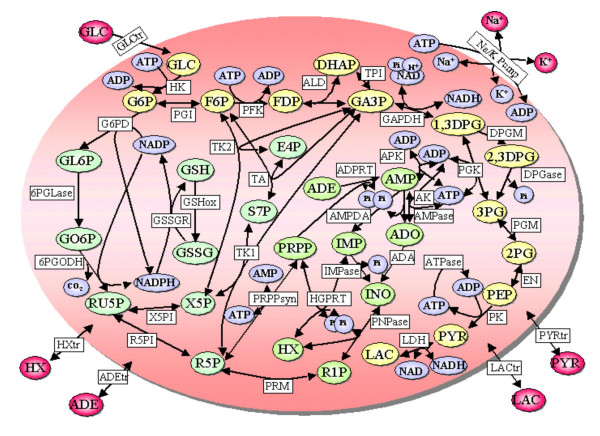
**Metabolic map of the prototype RBC model**. The circles are metabolic intermediates and ions. These molecular species are defined as "Substance" in the E-Cell system. The boxes are enzymes and reaction processes. Their rate expressions are defined as "Reactor" whereas the enzyme molecules are defined as "Substance".

The E-Cell system version 1.1 was used as the simulation platform in this work. The software can be downloaded from . Our prototype model of the RBC was developed on the basis of the whole-cell model of Joshi and Palsson [8-11] with slight modifications (Figure 1). We modified the model to represent the oxidant-induced decrease of hexokinase and pyruvate kinase, and the maximum activity of these enzymes was allowed to change according to the ratio of GSH and GSSG. The equations and parameters used are derived from the literature [17]. The parameters and kinetic equations in the original model of Joshi and Palsson were replaced with those obtained from the literature [17,20,21] (Table 1) in order to fit the model to the measured concentrations during the calculation of the steady state. The steady state obtained had concentrations of many metabolites that were very close to those in real RBCs (Table 2). However, the concentrations of several metabolites, namely adenosine, hypoxanthine, inosine, 5-phosphoribosyl 1-phosphate and ribose 1-phosphate, differed from the experimental values. These differences were due to the kinetic parameters and equations used, and because the nucleotide metabolism in the original model was represented as simple first-order kinetics or equilibrium.

**Table 1:** Enzymes and rate equations of the prototype model

**Enzymes**	**Abbreviation**	**Group**	**Reaction mechanism**	**Reference**
Glutathione turnover	GSHox	PPP	Chemical reaction	24
Glutathione reductase (NADPH)	GSSGR	PPP	Ordered Bi Ter mechanism	24
Glutathione reductase (NADH)	GSSGR2	PPP	Michaelis Menten mechanism	24
Glucose 6-phosphate dehydrogenase	G6PD	PPP	Ordered Bi Bi mechanism	17
6-Phosphogluconolactonase	6PGLase	PPP	Michaelis Menten mechanism	17
6-Phosphogluconate dehydrogenase	6PGLDH	PPP	Ordered Bi Ter mechanism	24
Ribose 5-phosphate isomerase	R5PI	PPP	Uni Uni mechanism	25
Xylulose 5-phosphate isomerase	X5PI	PPP	Uni Uni mechanism	25
Transketolase I	TK1	PPP	Ping-Pong Bi Bi mechanism	25
Transketolase II	TK2	PPP	Ping-Pong Bi Bi mechanism	25
Transaldolase	TA	PPP	Ping-Pong Bi Bi mechanism	25
Hexokinase	HK	Glycolysis		26
Phosphoglucoisomerase	PGI	Glycolysis	Uni Uni mechanism	25
Phosphofructokinase	PFK	Glycolysis		27
Aldolase	ALD	Glycolysis	Ordered Uni Bi mechanism	25
Triose phosphate isomerase	TPI	Glycolysis	Uni Uni mechanism	25
Glyceraldehyde phosphate dehydrogenase	GAPDH	Glycolysis	Chemical reaction	20
Phosphoglycerate kinase	PGK	Glycolysis	Chemical reaction	20
Diphosphoglycerate mutase	DPGM	Glycolysis	Michaelis Menten mechanism	20
Diphosphoglycerate phosphatase	DPGase	Glycolysis	Michaelis Menten mechanism	20
Phosphoglyceromutase	PGM	Glycolysis	Chemical reaction	20
Enolase	EN	Glycolysis	Chemical reaction	20
Pyruvate kinase	PK	Glycolysis		28
Pyruvate transport process	PYRtr	Transport	Michaelis Menten mechanism	22
Lactate dehydrogenase	LDH	Glycolysis	Chemical reaction	20
Lactate transport process	LACtr	Transport	Michaelis Menten mechanism	22
Leak of Potassium	K_Leak	Transport		9
Leak of Sodium	Na_Leak	Transport		9
Sodium/potassium pump	Pump	Transport		9
Adenosine transport process	ADEtr	Transport	Chemical reaction	13
AMP phosphohydrolase	AMPase	NM	Chemical reaction	20
Adenosine deaminase	ADA	NM	Michaelis Menten mechanism	20
Adenosine kinase	AK	NM	Michaelis Menten mechanism	20
Adenylate kinase	APK	NM	Chemical reaction	20
Adenosine triphosphate phosphohydrolase	ATPase	NM	Chemical reaction	8
Adenosine monophosphate deaminase	AMPDA	NM	Michaelis Menten mechanism	20
Inosine monophosphatase	IMPase	NM	Michaelis Menten mechanism	8
Purine nucleotide phosphorylase	PNPase	NM	Chemical reaction	23
Phosphoribosyl pyrophosphate synthetase	PRPPsyn	NM		8
Adenine phosphoribosyl transferase	ADPRT	NM	Michaelis Menten mechanism	8
Hypoxanthine-guanine phosphoryl transferase	HGPRT	NM	Michaelis Menten mechanism	8
Hypoxanthine transport process	HXtr	NM		29

PPP, Pentose phosphate pathway; NM, Nucleotide metabolism.

**Table 2:** Steady state of the RBC model.

		Concentration (mM)	
		
Metabolic intermediate	Abbreviation	Steady state^b^	Literature^c^
1,3-Diphosphoglycerate	13DPG	1.83E-04	4.00E-04
2-Phosphoglycerate	2PG	4.16E-03	1.40E-02 ± 5.00E-03
3-Phosphoglycerate	3PG	4.62E-02	4.50E-02
Adenosine	ADO	8.93E-06	1.20E-03 ± 3.00E-04
Dihydroxy acetone phosphate	DHAP	1.35E-01	1.40E-01 ± 8.00E-02
Erythrose 4-phosphate	E4P	1.17E+00	-
Fructose 6-phosphate	F6P	6.39E-02	1.60E-02 ± 3.00E-03
Fructose 1,6-diphosphate	FDP	1.14E-02	7.60E-03 ± 4.00E-03
Glucose 6-phosphate	G6P	1.96E-01	3.80E-02 ± 1.20E-02
Glyceraldehyde 3-phosphate	GA3P	6.24E-03	6.70E-03 ± 1.00E-03
Gluconolactone 6-phosphate	GL6P	7.62E-06	-
Gluconate 6-phosphate	GO6P	2.72E+00	-
Glutathione	GSH	3.21E+00	3.21E+00 ± 1.50E+00
Glutathione	GSSG	1.03E-04	-
Hypoxanthine	HXi	9.32E-06	2.00E-03
Inosine monophosphate	IMP	5.03E-03	1.00E-02
Inosine	INO	3.32E-08	1.00E-03
Potassium	Ki	1.26E+02	1.35E+02 ± 1.00E+01
Lactate	LACi	1.20E+00	1.10E+00 ± 5.00E-01
Nicotinamide adenine dinucleotide	NAD	8.87E-02^d^	-
Nicotinamide adenine dinucleotide	NADH	3.13E-04^d^	-
Nicotinamide adenine phosphate	NADP	8.06E-05^d^	-
Nicotinamide adenine phosphate	NADPH	6.58E-02^d^	6.58E-02
Sodium	Nai	2.27E+01	1.00E+01 ± 6.00E+00
Phosphoenolpyruvate	PEP	1.89E-02	1.70E-02 ± 2.00E-03
5-Phosphoribosyl 1-phosphate	PRPP	6.91E-05	5.00E-03 ± 1.00E-03
Pyruvate	PYRi	6.00E-02	7.70E-02 ± 5.00E-02
Inorganic phosphate	Pi	1.30E-01	1.00E+00
Ribose 1-phosphate	R1P	2.12E-05	6.00E-02
Ribose 5-phosphate	R5P	2.81E-04	-
Ribulose 5-phosphate	RU5P	1.48E-04	-
Sedoheptulose 7-phosphate	S7P	7.49E-02	-
Xylulose 5-phosphate	X5P	4.30E-04	-
2,3-Diphosphoglycerate	2,3-DPG	4.21E+00	4.50E+00 ± 5.00E-01
Adenosine diphosphate	ADP	2.20E-01	2.70E-01 ± 1.20E-01
Adenosine monophosphate	AMP	2.42E-02	8.00E-02 ± 9.00E-03
Adenosine triphosphate	ATP	1.57E+00	1.54E-00 ± 2.50E-01

The values are given in scientific notation; E-01 denotes multiplication by 10^-1^.

^a^The initial data set was from experimental data in the literature and from predictions of previous simulation models [12].

^b^The simulation was run for more than 1,000,000 seconds in simulation time until the model reached steady state.

^c ^Biochemical experimental data taken from the literature and reported in Joshi and Palsson [11].

^d ^NAD(H) and NADP(H) pools are kept constant.

The parameters from the work of Jacobasch *et al*. [30] were used in the experiments simulating G6PD deficiency (Table 3). Since the rate equation of G6PD deficiency is the same as that in the normal cell, the parameters were simply replaced in the deficiency experiment. We adopted the We.G variant of G6PD deficiency because its parameters are well described in the literature and its phenotype is rather severe. As with the original model, the oxidative load is represented as the conversion of GSH to GSSG, and the equation is expressed as a simple first-order kinetics.

**Table 3:** Parameters for normal and deficient enzymes

	t/2 (day)	Vmax (mkat/l cells)	KmG6P	KmNADP (mM)	KiNADPH	KiATP	Ki2,3DPG
Normal	27	575	67	3.7	3.1	749	2289
We.G.	2.5	10	152	3.8	0.62	180	520

These values are based on experimental data taken from the literature [10]

### Expansion of the prototype model and simulation experiments

The *de novo *GSH synthesis and GSSG export pathways (Figure 3) were added to the prototype model. The kinetic equations and parameters of these pathways were obtained from the literature [31-33] (Table 4). Since these pathways have very low activity in normal cells, the concentrations of metabolites at the steady state were almost unchanged in the expanded model. The concentrations listed in Table 2 were used as the steady state concentrations. The conditions employed to simulate G6PD deficiency using this expanded model were the same as those of the prototype model. It is known that multidrug resistance-associated proteins (MRP1) and the cystic fibrosis transmembrane conductance regulator (CFTR) are expressed in human RBC and involved in GSH and/or GSH conjugates transport [35]. However, their rate equations and parameters are unavailable, so these proteins were not included in this model.

**Figure 2:**
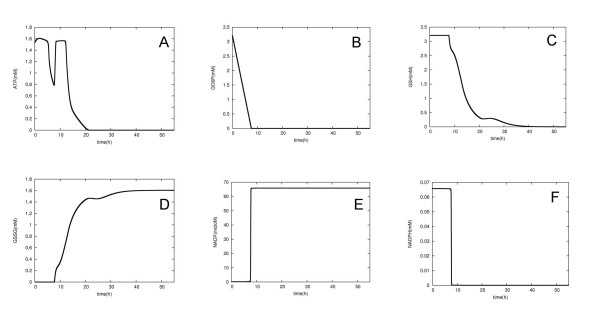
**Pathway for the *de novo *of GSH and the GSSG export system**. *γ*-GCS, *γ*-glutamyl cysteine synthetase; *γ*-CS, *γ*-glutamyl cysteine.

**Table 4:** Rate equations and parameters of GSH synthesis and GSSG export that were used in the expanded model.

Rate equation for *γ*-glutamyl cysteine synthetase		

Parameters for *γ*-glutamyl cysteine synthetase		
Parameter	Value	Reference
Vmax	141.57 mM/h	31, 32
*α*	0.2	31
Km*glu*	1.8 mM	31
Km*cys*	0.1 mM	31
Ki*GSH*	3.4 mM	31
Km*ATP*	0.4 mM	31
		
Rate equation for glutathione synthetase		


Parameters for glutathione synthetase		

Parameter	Value	Reference
Km_*γ*_GC_	0.99 mM	33
Km_Gly_	1,37 mM	33
Km_ATP_	0,23 mM	33
*α*	2.6	33
Vmax	88.4 mM/h	33
		
Rate equation for GSSG export		


Parameters for GSSG export		

Parameter	Value	Reference
Km_GSSG1_	0.1 mM	34
Km_ATP_	0.63 mM	34
Vm_1_	20 *μ*M/h	34

## Results and Discussion

### Simulation of G6PD deficiency using the prototype model

The prototype model was used to simulate the effects of G6PD deficiency. G6PD is a key enzyme in the pentose phosphate pathway that converts glucose 6-phosphate into gluconolactone 6-phosphate (GL6P); this simultaneously generates NADPH. The metabolic intermediate GL6P is then metabolized into ribulose 5-phosphate (Ru5P) acid *via *gluconate 6-phosphate (GO6P). This process also generates NADPH. This reduction power is employed by various other intracellular processes, in particular the reduction of GSSG. A major function of GSH in the RBC is to eliminate superoxide anions and organic hydroperoxides. Peroxides are eliminated through the action of glutathione peroxidase, which yields GSSG.

The simulation experiments were carried out with steady state concentrations corresponding to those in the normal RBC. Sequential changes in the quantities of NADPH, GSH and ATP were observed (Figure 2). There is a negative peak in ATP concentration before 10 h. This was due to the shutting down of the pentose phosphate pathway. The Ru5P produced was mainly converted to fructose 6-phosphate (F6P), and this metabolite consumed ATP to make fructose 1,6-diphosphate (FDP). The FDP production led to an accumulation of dihydroxy acetone phosphate (DHAP), and the metabolite was not used to provide ATP. The high GO6P concentration could sustain normal levels of GSH concentration at the first stage of the simulation, but after the depletion of GO6P the rate of Ru5P production was drastically reduced. This decrease in Ru5P concentration led to decreased F6P concentrations.

**Figure 3:**
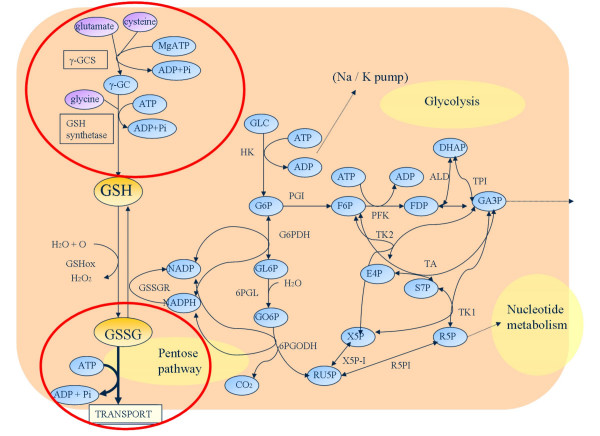
**Computer simulation time-course of metabolic intermediates**. Changes in the concentrations of ATP (A), GO6P (B), GSH (C), GSSG (D), NADP (E) and NADPH (F) during the RBC simulation. The simulation was run for 200,000 seconds (Approx. 55 h) in simulation time. Concentrations change when G6PD kinetic parameters are shifted from the normal to pathological values (Table 3). ATP became depleted at around 20 h.

At around 20 h, ATP was rapidly consumed and depleted. Since ATP concentrations less than half the normal concentration have never been observed in enzyme deficiencies [36], cells in this condition will probably be destroyed. Although the half-life of the real G6PD-deficient We.G type RBC is known to be 2.5 days [30], the longevity of our computer model turned out to be much shorter (Table 3). Since data on the concentration of metabolites in RBCs with G6PD deficiency are not available, it was not possible to determine whether the metabolite concentrations arising in our simulation experiments reflected those observed in real cells.

### Simulation of G6PD deficiency using the expanded model

It is obvious that decreased pentose phosphate pathway activity leads to faster cell death, and that the difference between the simulated cell and the real cell regarding the timing of cell death could be caused by the lack of a pathway producing GSH. This pathway may compensate for the decrease in GSH. A mature RBC normally contains 2 mM GSH but contains only several *μ*M GSSG. Although GSSG reductase plays a prominent role in maintaining a stable GSH/GSSG ratio, other processes, including *de novo *GSH synthesis and GSSG export pathways, may generate GSH in the G6PD-deficient cell.

**Figure 4:**
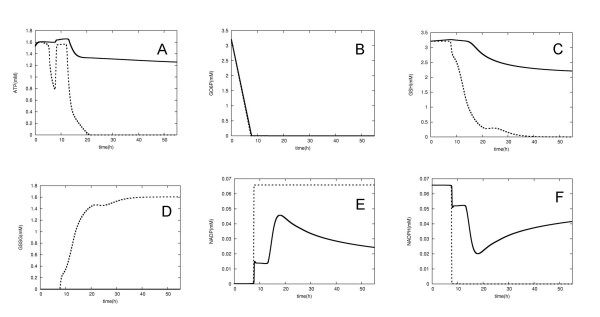
**Simulation of G6PD deficiency using the expanded model**. Changes in the concentrations of ATP (A), GO6P (B), GSH (C), GSSG (D), NADP (E) and NADPH (F) during RBC simulation. Broken lines are the results of the prototype model, while solid lines are the results of the expanded model during the same parameter shift as described in Figure 2. The simulation was run for 200,000 seconds (Approx. 55 h) in simulation time.

**Figure 5:**
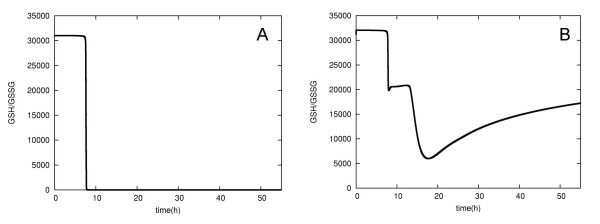
**The GSH/GSSG ratio of the prototype and expanded models**. The prototype model (A) and the expanded model (B).

After the expansion of the prototype model to include *de novo *GSH synthesis and GSSG export, the ATP levels were maintained at 80% of normal and the cell was longer lived (Figure 4). In addition, the GSH/GSSG ratio was higher (Figure 5). This indicates that the *de novo *GSH synthesis pathway can partially compensate for the lowered GSH concentrations resulting from G6PD deficiency, and that the concentration of GSSG can be kept at a very low level due to the active export system. These observations suggest that these reactions could alleviate the anemia resulting from G6PD deficiency. It is known that people with this deficiency are not normally anemic and display no evidence of the disease until the RBCs are exposed to oxidant stress. The compensatory effect of the *de novo *GSH synthesis and GSSG export pathways may thus help to explain why many varieties of G6PD deficiency have no evident phenotype. Moreover, it has been proposed that the high frequency of G6PD deficiency may be due to its ability to protect against malaria. Our observations suggest that the compensatory mechanism we have elucidated may have aided this spread of G6PD deficiency, as it counterbalances the worst effects of the deficiency, thus decreasing its severity and promoting the propagation of the disease during evolution.

### Determination of a range of metabolic pathways for modeling

These results showed that the *de novo *GSH synthesis pathway and the GSSG export system are essential for accurate simulation of G6PD deficiency in human RBCs. Previous simulations of this deficiency have not included these pathways [17] and the results they generated were similar to those obtained using our prototype model (Figure 2). Our prototype model and the previous models developed by others contain only three metabolic pathways, namely, the glycolysis pathway, the pentose phosphate pathway and the nucleotide metabolic pathway. Although these models are sufficient for representing the normal state of the human RBC, they are not adequate for simulating irregular conditions such as deficiencies, because they lack alternative pathways that may normally not be particularly active but can compensate for the deficiency to some extent. Indeed, our results indicate that all the metabolic pathways in the cell will be needed to develop a general purpose model that can be used to simulate any condition. However, dynamic simulation based on kinetic equations requires a large variety of rate equations and kinetic parameters, and unfortunately, such data are rarely available as a complete set. Recently, our laboratory proposed a novel simulation method that reduces the need for this kind of information [37]. This hybrid dynamic/static simulation method combines dynamic rate equations with a flux-based approach and as a result reduces the numbers of rate equations and parameters that are needed by up to 70–80%. It may solve the problems associated with developing a model that simulates all the cellular metabolic pathways.

### The mathematical steady state may not be the normal state of real cells

During this simulation analysis, we realized that the longevity of enzymes should be considered in long-term simulation experiments. While in our model the activities of enzymes are decreased by oxidants, enzymes also generally become degraded over time. This natural decrease is not included in our model. As shown in this work, the prototype model was able to achieve a steady state. However, this mathematical steady state, which is when the rates of the production and consumption of all metabolic intermediates become equal, may not exactly represent the condition of the RBCs in the human body. Such a "mathematical steady state" never occurs in living organisms, especially in higher multicellular organisms. Rather, homeostasis in multicellular organisms is maintained by replacing the loss of disposable cells with additional cells. It is possible that these disposable cells never reach a mathematical steady state. Thus, a model that can tolerate long-term simulation for analyzing the pathology of human diseases should not approximate the "mathematical steady state". Moreover, in the case where the system reaches a steady state with a certain oscillation, it is impossible to obtain a mathematical steady state using an accurate model. It is known, for example, that some key enzymes in glycolysis bind to the Band III protein, an abundant membrane protein in the human RBC [38-40]. The interaction between glycolytic enzymes and Band III varies depending on the ratio of oxyhemoglobin to deoxyhemoglobin, and it is believed that this interaction is responsible for some oscillations in metabolic pathways in the human RBC.

## Conclusion

We developed a computer model of the human RBC that is based on a previous model but was expanded by introducing a GSH synthesis pathway and a GSSG export system. With this expansion, the model maintained high ATP concentrations in G6PD deficiency. This suggests that these pathways may play an important role in alleviating the consequences of G6PD deficiency. It also indicates that sub-pathways that are normally not particularly highly activated may play important roles in abnormal conditions such as deficiencies.

## Authors' contributions

Nakayama contributed mostly to the model development, Kinoshita contributed to the analysis, and Tomita developed the basic ideas and directed the project.

## Competing interests

The author(s) declare that they have no competing interests.
